# Post-Mortem Interval and Microbiome Analysis through 16S rRNA Analysis: A Systematic Review

**DOI:** 10.3390/diagnostics12112641

**Published:** 2022-10-31

**Authors:** Pamela Tozzo, Irene Amico, Arianna Delicati, Federico Toselli, Luciana Caenazzo

**Affiliations:** Department of Cardiac, Thoracic, Vascular Sciences and Public Health, University of Padova, 35121 Padova, Italy

**Keywords:** PMI (Post-Mortem Interval), microbiome, 16S rRNA, forensic science, systematic review

## Abstract

The determination of the Post-Mortem Interval (PMI) is an issue that has always represented a challenge in the field of forensic science. Different innovative approaches, compared to the more traditional ones, have been tried over the years, without succeeding in being validated as successful methods for PMI estimation. In the last two decades, innovations in sequencing technologies have made it possible to generate large volumes of data, allowing all members of a bacterial community to be sequenced. The aim of this manuscript is to provide a review regarding new advances in PMI estimation through cadaveric microbiota identification using 16S rRNA sequencing, in order to correlate specific microbiome profiles obtained from different body sites to PMI. The systematic review was performed according to PRISMA guidelines. For this purpose, 800 studies were identified through database searching (Pubmed). Articles that dealt with PMI estimation in correlation with microbiome composition and contained data about species, body site of sampling, monitoring time and sequencing method were selected and ultimately a total of 25 studies were considered. The selected studies evaluated the contribution of the various body sites to determine PMI, based on microbiome sequencing, in human and animal models. The results of this systematic review highlighted that studies conducted on both animals and humans yielded results that were promising. In order to fully exploit the potential of the microbiome in the estimation of PMI, it would be desirable to identify standardized body sampling sites and specific sampling methods in order to align data obtained by different research groups.

## 1. Introduction

Estimating the time since death has always represented a challenge for forensic sciences, as establishing the post-mortem interval (PMI) is one of the main objectives of the forensic pathologist in real casework.

Classically, the thanato-chronological assessment of PMI is based on the correct interpretation of the three classic signs of consecutive abiotic cadaveric phenomena [1]: thermal decrement (algor mortis), hypostasis formation (livor mortis) and cadaveric stiffening (rigor mortis). The assessment of post-mortem physical changes is usually used for estimating early PMI, i.e., within the first 24 h post-mortem. These criteria, still widely used in daily practice and real casework by forensic pathologists, may present some weaknesses, such as the fact that they may be influenced by endogenous and exogenous factors, the fact that they are operator-dependent and that, even when registered with the utmost expertise, they are only able to provide information that circumscribes the time of death in a more or less wide time interval, but with a fair degree of uncertainty.

During recent decades, several innovative methods used in other clinical and research fields were tested as being able to provide a higher degree of specificity in PMI estimation [2]. These studies may range from analysing ionic concentration in bodily fluids [3], to investigating degradation patterns of macromolecules (proteins, RNA and DNA) [4,5,6,7], to the analysis of metabolites in the organism [8], to applying radiological technologies [9,10,11], to perform immune-histochemical (IHC) tests to evaluate tissues’ morphological changes [12,13], to entomological analysis in the more advanced decomposition stages [14], and to the change in body heat emission [15].

Each method proposed in the literature has proved to be in some way useful, with different strengths but also with some limitations, both of a technical-executive nature and of an interpretative nature, given that they all seem to be influenced by environmental and cadaveric factors [16]; the gold standard for determining the PMI cannot yet be reduced to a single method [17,18]. One of the limitations of the various PMI estimation methods is that each of them is able to investigate a more or less specific period. In addition, it would be desirable to be able to probe the same time interval with different methods in order to increase the precision of the PMI estimation.

In recent years, a great deal of research has taken place to demonstrate the role that the microbiome analysis of cadaveric samples could play in the forensic field [19,20,21,22,23].

Microbiota is defined as a very large community in which there are single-celled bacteria, viruses, fungi, archaea and eukaryotes, while microbiome represents the set of genetic heritage and interactions of the previously mentioned organisms within our organism. Being a dynamic entity, human microbiota undergoes important changes during human life in response to numerous environmental and individual factors, as for example smoking habits, drug treatments, diet and healthcare status [24,25,26]. The spectrum of ways that microbiome could be used is wide [27,28,29,30,31,32]. From this point of view, microbiota can be defined as a meta-organ, i.e., a structure that is anatomically not part of our organism, but which, having evolved with man, takes part in it and is fundamental for its correct physiology in life, possibly playing a role in the post-mortem decomposition stages and PMI estimation [33].

However, carrying out studies on the microbiome of samples taken from human cadavers under standardized conditions is not easy for several reasons, such as the limited availability of cadavers, the difficulty of standardizing factors that influence the microbiome (in particular the cause of death, age, health conditions) and the high cost of the sequencing techniques that are used [34].

All members of a bacterial community can be sequenced using various sequencing approaches: massively parallel sequencing (MPS), long-read sequencing and Sanger sequencing may be used, allowing a high-resolution determination of the composition of the microbiome.

Nowadays, the main options supplied by next generation sequencing (NGS) techniques for microbiome sequencing are essentially two, which, except for the first steps, have separate paths: these strategies are meta-genomic shotgun sequencing and amplicon sequencing. The first one gives the opportunity to analyse, at the same time, the whole number of DNA fragments found in a sample [35].

The anticipated second approach, that is amplicon sequencing, is rather different from the previous one: instead of considering all the DNA within a sample, it targets 16S rRNA genes, which are typically selected as taxonomic marker genes (the so called “amplicon”) for bacteria or archaea. This gene encodes for the small subunit of the bacterial ribosome and possessing variable regions makes it possible to define phylogenetic relationships. These genes are approximately 1550 base pairs long and consist of eight highly conserved regions and nine extremely species-specific hypervariable regions [36]; the latter feature makes them fit to create operational taxonomic units (OTUs) made of the most alike sequences, permitting definition of the phylogenetic relationships by confronting the OTUs and, eventually, characterization of the composition of the bacterial community [37]. OTUs have replaced “species” in many microbiome analyses since named species’ genomes are often unavailable for particular marker sequences [38]. A great strength of this sequencing technique is that a phylogenetic classification can sometimes be made from fragments as small as 100 bp of 16S rRNA, making microbiome analysis even more affordable [39]. Moreover, in comparison with the whole genome sequencing approach, focusing only on certain regions reduces the risk of sample damaging.

Examination of human microbiomes through directed sequencing of 16S ribosomes (rRNA genes) allows for an estimation of the taxonomic diversity and the distribution of the contributory bacterial species, since the targeted 16S rRNA sequencing can be analysed bioinformatically, having the advantage of high-quality databases, reliable classification pipelines for sequencing data and the possibility of a greater read depth and a reduced cost when compared with metagenomic sequencing [40]. The 16S ribosomal RNA (or 16S rRNA) is the RNA component of the 30S subunit of a prokaryotic ribosome (SSU rRNA- Small subunit ribosomal ribonucleic acid). Two parameters can be used to describe the microbial variability of a given sample: alpha-diversity is the observed richness (number of taxa) or evenness (the relative abundances of those taxa) of an average sample within a habitat type, while beta-diversity is the variability in community composition (the identity of taxa observed) among samples within a habitat [41].

The objective of this review is to analyze the existing literature on the role of the cadaveric microbiome, determined with the study of 16S rRNA, in the estimation of PMI.

## 2. Materials and Methods

This review was performed in adherence to the Preferred Reporting Items for Systematic Reviews and Meta-Analyses (PRISMA) 2020 guidelines [42].

A systematic literature review of English and non-English papers regarding estimation of post-mortem interval correlated with microbiome composition was conducted by two authors (I.A. and F.T.) using public electronic databases (PubMed). The research strategy included the terms “PMI”, “forensic” and “microbiome” in the following combinations: “forensic (and) microbiome” and “PMI (and) microbiome”. The search terms were intentionally kept generic in order to include all potentially interesting papers about the topic.

A total of 800 works were identified through database searching. Duplicates (40 works) were removed manually. Articles were screened using filters (A) English language; (B) Date of publication, i.e., articles published from 2000 to 2022. A total of 750 articles published from 2000 to 2022 were thus selected. Then, the two authors, independently of each other, performed a first selection of the remaining articles according to the inclusion criteria: (C) only articles dealing with estimation of post-mortem interval correlated with microbiome composition. Then, all works that did not provide the following data (i.e., investigation model, human or animal, and, in the latter case, the animal species under consideration, stated the body site of the sampling; specified monitoring time, described the sequencing method) were excluded (D). We first screened titles for inclusion criteria, then abstracts, and only, when necessary (i.e., the topic was not clear from the title and/or abstract reading), did the authors undertake a full-text evaluation. In cases of disagreement or further doubts, the supervisors (L.C. and P.T.) were queried.

After title and abstract evaluation, 700 articles were excluded due to irrelevance. After a full text reading of the selected papers, only 25 were considered eligible using criteria and included in the review. For each article, the authors examined the full text and extracted the following data, managing them in Excel^®^ (Microsoft^®^ 365): reference (author and year of publication), model, body site, numerosity, temperature and time interval.

**Figure 1:**
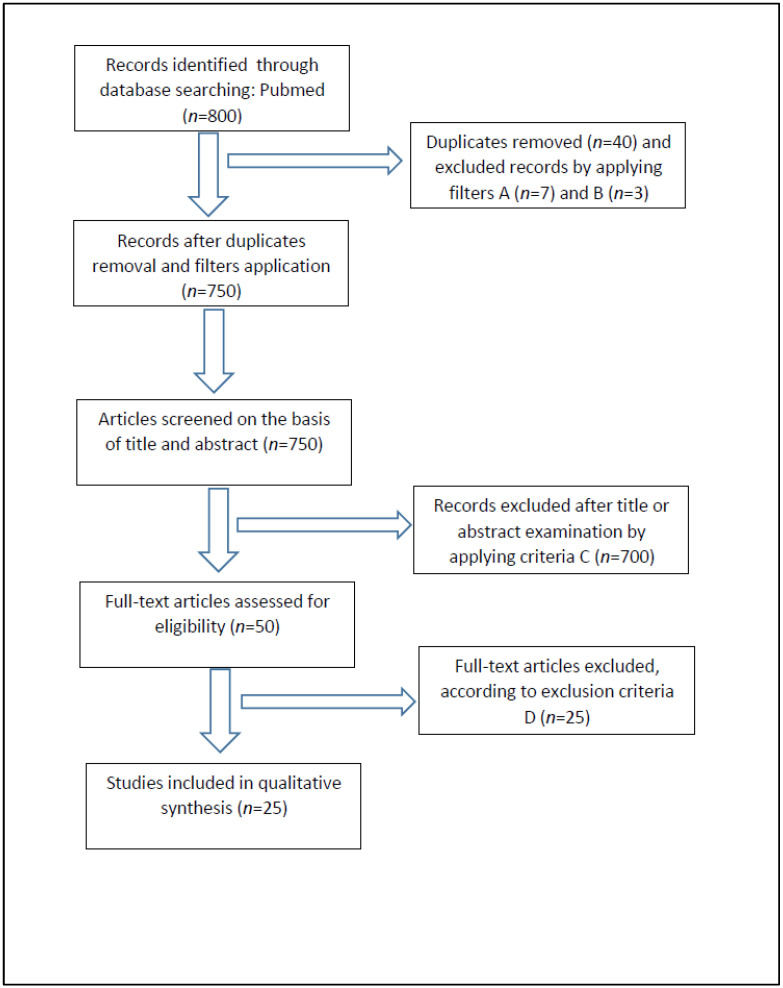
PRISMA 2020 flow diagram for new systematic reviews which included searches of databases and registers only.

The PRISMA flow chart in Figure 1 summarizes the study screening and selection process as described above.

## 3. Results

From 2000 to 2022, several studies demonstrated a correlation between the composition of microbiome and PMI. These studies are summarized, in chronological order, in Table 1, in which we have specified, for each study, the experimental model used (animal or human), the body site of sampling, the sample size, the temperature and the time interval of experimental observation.

According to the model used, we subdivided the experimental articles into categories: one composed of 11 articles with animal models, one composed of 13 articles with human models and one with only one study using both models. Furthermore, in order to give a more understandable reading, articles were subcategorized based on the body site of the sampling as follows:

- Sites communicating with the outside: possible sampling sites without the need for incision/biopsy, i.e., eyes, ears, nose, mouth, rectum and skin.
- Internal sites: sampling requiring incision/biopsy, i.e., brain, spleen, liver, heart, prostate, uterus and bones.
- Both external and internal sites.
- Not only body sampling (e.g., grave soil).

### 3.1. Animal Models

According to what has been previously described, we based the subdivision of the articles on the body site of sampling, finding three articles that consider external sampling sites, six internal sampling sites and two not only body sites.

#### 3.1.1. Animal Model-Sites Communicating with the Outside

In 2016, Guo et al. [43] analysed bacterial communities from live rats and rat carcasses (*n* = 18), using Illumina MiSeq sequencing of 16S rRNA gene amplicons. Bacterial communities were sampled prior to death, immediately after death and then at 4 h, 12 h, 1 day, 2 days, 3 days, 4 days, 6 days and 8 days after death. They noticed that bacterial communities showed variation in relative abundance and became more like each other across body sites during decomposition processes. In oral cavity, Proteobacteria became the predominant phylum while Firmicutes gradually decreased as decomposition progressed. Likewise, in the rectum Proteobacteria increased while Bacteroidetes gradually decreased with decomposition.

In 2019, Dong et al. [44] observed that the oral bacterial community showed changes in relative abundance during the decomposition process of 24 adult mice within 240 h after death. In addition, they found that, at different taxonomic levels, specific bacteria were found to be significantly correlated with PMI. By creating linear regression models between relative abundance and PMI, they achieved that, to infer the PMI, *Gammaproteobacteria* and *Proteus* were the best species to be considered.

In 2021, Li et al. [45] investigated the probable shift in the composition of the rectal microbiota in eight rats at different time intervals up to 15 days after death to explore important taxa for estimating PMI. They discovered that the turning point occurred at day 9 when the microbiome provided the most useful information for estimating the time since death. They constructed a prediction model using data from high-throughput sequencing and seven bacterial taxa were included in this model.

#### 3.1.2. Animal Model-Internal Anatomical Sites

In 2012, Heimesaat et al. [46] carried out a kinetic survey of naturally occurring post-mortem gut flora changes in the bowels of conventional and gnotobiotic mice associated with a human microbiota (HFA) applying cultural and molecular methods.

Fresh human faecal samples for recolonizing HFA mice were collected from healthy volunteers. 3 months old female mice were sacrificed and kept at constant standardised external conditions (21 °C, 50% relative humidity). Intestinal and extraintestinal samples were immediately removed from each mouse within 5 min in parallel for microbiological analysis under sterile conditions at the respective post-mortem time point. For qualitative detection of bacterial translocation, organs were each transferred into a thioglycolate broth and incubated for maximum 7 days at 37 °C. Bacterial growth was monitored daily through quantitative RT-PCR analyses by specific amplification of the 16S rRNA genes of up to nine main bacterial groups. They found that intestinal microbiota changes were most pronounced in the ileal lumen, where *enterobacteria* and *enterococci* increased by 3–5 orders of magnitude in conventional and HFA mice. In HFA mice, ileal overgrowth with *enterococci* started 3 h post-mortem, and with *enterobacteria* 24 h post-mortem. They concluded that the kinetic of ileal overgrowth with *enterococci* and *enterobacteria* in HFA mice could be used as an indicator to define more precisely the time point of death under specific ambient conditions.

In 2018, Iancu et al. [47] published a work that aimed to assess the diversity and dynamics of bacterial changes throughout the decomposition stages of 60 buried rat carcasses in a natural environment for 30 days, sampling specimens from the small intestine. The results of this study provided further insights into decomposition and contributed to a greater understanding of the microbiological factors involved in decomposition by analysing microbiome qualitatively and quantitatively.

In 2019, Burcham et al. [48] monitored bacterial community changes occurring during decomposition of heart, bone marrow, lungs and intestines in a murine model. They found out that organs presumed to be sterile during life were colonised by *Clostridium* during later decomposition. They created a model that reinforced the known data on bacterial taxonomic succession after death. This study is one of the first to provide data on genes expressed by the bacterial community. The same main author in the same year conducted another study [49] using murine models, sampling internal organs (i.e., bone marrow, heart, intestines and stomach), which demonstrated the use of meta = transcriptomics from the post-mortem microbiome to obtain the composition of the microbiota community in PMI. Further, they found that bacterial succession patterns showed similar trends to those detected through DNA analysis.

In 2020, Liu et al. [50] performed a study in which they combined microbial community characterization, microbiome sequencing from different organs (i.e., brain, heart and cecum) and machine learning algorithms to investigate microbial succession pattern during corpse decomposition, in order to estimate PMI in murine model. Significant differences in microbial communities’ composition were found between the time of death and advanced decomposition stages. They found that the artificial neural network model combined with the post-mortem microbiome data set from the cecum was the best combination to be used, finding a mean absolute error of 1.5 ± 0.8 h within 24 h decomposition and 14.5 ± 4.4 h within 15 days decomposition.

Finally, the same author [51] in 2021 published a study that used hierarchical clustering based on Manhattan distances to analyze the similarities and differences among post-mortem intestinal (cecum) microbial succession patterns. The normal intestinal flora in the cecum decreases after death, while some facultative anaerobes and obligate anaerobes increase. They developed a random forest regression model to predict the PMI based on the microbial succession trend dataset, with a mean absolute error of 20.01 h during the first 15 days of decomposition.

#### 3.1.3. Animal Model—Not Only Body Samples

In 2013, Metcalf et al. [52] performed an experiment in which 40 mice were allowed to decompose after death on soil graves. They sampled the microbial communities from the mouse abdomen and skin, as well as from the associated grave soil, from five replicate corpses over eight time points that spanned 48 days with sampling events occurring more frequently (every 3 days) over the first 2 weeks. For sequencing, total genomic DNA was subjected to PCR amplification targeting an informative portion of the 16S rRNA VR4 using a bacterial primer. They discovered that temporal changes in microbial communities of the head skin allowed them to estimate the PMI within 3.30 ± 2.52 days over 48 days.

In 2021, Zhang et al. [53] published a work that focused on buried corpses. They characterised the microbial communities from grave soil, rectum and skin of 50 burial rat cadavers for 60 days, using 16S rRNA gene high-throughput sequencing. They found that later decay stages had a lower alpha-diversity compared to earlier post-mortem stages. Significant linear relationships between similarities of the microbial communities and PMI were observed. They combined random forest models with composition of the microbiome to predict PMI. The model explained in percentages higher than 80% of the variation in microbial community with a mean absolute error of less than 2.13 days within 60 days of decomposition, with lower error in grave soil analysis than in rectum or skin ones.

### 3.2. Human Models

Using the same subdivision criterion applied in Section 3.1, we found four articles that consider external sampling sites, five internal sampling sites, two both external and internal sampling sites and two not only body sites.

#### 3.2.1. Human Model-Sites Communicating with the Outside

In 2016, Johnson et al. [54] sampled 21 decomposing cadavers, focusing on the nasal and ear canal microbiota. They developed several models of statistical regression to establish an algorithm for predicting the PMI of microbial samples. They found that the complete data set was preferred for training the regressor, instead of a curated list of indicator species. They developed a k-nearest-neighbour regressor that predicted the PMI of unknown samples with an average error of ±55 accumulated degree days (i.e., a unit that represents the amount of time that an organism spends at a temperature above its lower development threshold and below its upper development threshold; this is used as a projection of organism development).

In 2017, Adserias-Garriga et al. [55] published a study to monitor the microbiome of decaying bodies on a daily basis to identify signature bacterial taxa. They daily sampled the oral cavity of three human cadavers at different stages of putrefaction. Microbial DNA was extracted and analysed by NGS techniques, showing similar succession changes among different corpses throughout decomposition process.

In 2018, Kodama et al. [56] studied changes in skin microbiome of 16 cadavers for a period of 60 h after death. They found that post-mortem skin microbiomes were stable during repeated sampling up to 60 h and were similar to microbiomes of ante-mortem control human samples. In addition, alpha diversity was significantly higher for post-mortem skin microbiomes compared to ante-mortem ones.

Two years later, Pittner et al. [57] presented the first multi-methodological assessment of human post-mortem decomposition carried out on two buried body donors in Europe. In this study, the microbial community structure was not significantly different among sample types present at all dates and even when body sites were pooled there still was no community difference among different sampling dates. Random forest prediction accuracy among different sample site microbial communities was poor, with an error rate of 83.33% when predicting body site. However, random forest modelling was able to distinguish among sampling time-points with a 5.6% error rate without accounting for differences among donors.

#### 3.2.2. Human Model-Internal Anatomical Sites

In 2015, Hauther et al. [58] evaluated human gut bacterial populations to determine quantifiable time-dependent post-mortem changes. They sampled the proximal large intestine (cecum) of 12 human cadavers as they decayed under environmental conditions. The quantification of intestinal bacteria was performed by quantitative PCR using group-specific primers targeting 16S rRNA genes, concluding that *Bacteroides* and *Lactobacillus* abundances could be used as a reliable quantitative indicator of PMI.

In 2017, DeBruyn et al. [59] performed a study with the purpose of documenting post-mortem changes in human gut bacterial communities. They repeatedly sampled caecum of four human cadavers during their decay under natural environmental conditions. They found that bacterial richness significantly increased while diversity decreased, and the composition of gut bacterial communities changed similarly among different bodies over time towards a common decay community.

Some years later, in 2020, Lutz et al. [60] investigated microbiome variation among different organs and the extent to which microbial associations among different body organs in human cadavers can be used to predict PMI. They assessed microbiota of organ tissue (brain, heart, liver, spleen, prostate and uterus) of 40 cadavers with times of death ranging from 24 to 432 h. They found that uterus and prostate had a significantly higher alpha diversity than other anatomical sites and a significantly different microbial community composition from non-reproductive organs.

In 2021, Deel et al. [61] characterised the human bone microbial community in human cadavers. In particular, they analysed six human cadavers decomposing on the soil. Once ribs were exposed through natural decomposition, a rib was collected from each cadaver for eight time points at 3 weeks apart. They found a core bone microbiome dominated by taxa in the phylum Proteobacteria. They used the microbial community data to develop random forest models that predict PMI with an accuracy of approximately ±34 days over a 1- to 9-month time frame of decomposition.

Finally, in the same year, Hu et al. [62] collected gut samples from the vermiform appendix and the transverse colon of 63 human cadavers with PMIs between 5 and 192 h in order to determine their microbiota. Their results revealed that the appendix might be an excellent intestinal sampling site and that the microbial flora of the appendix follows a rule of succession throughout corpse decomposition.

#### 3.2.3. Human Model-Sites Communicating with the Outside and Internal Anatomical Sites

In 2013, Hyde et al. [63] published a study that analysed microbiome changes during post-mortem decomposition. They sampled various body sites including viscera of two cadavers at two time points: at the onset and end of the bloat stage of decomposition under natural conditions. By sequencing the 16S rRNA gene, they obtained data that show a shift from aerobic bacteria to anaerobic bacteria in all body sites sampled and demonstrated variation in community structure between bodies, sample sites within a body and between initial and end points of decomposition.

Three years later, Javan et al. [64] reported a study of the sampling of 27 human corpses with PMI comprised between 3.5–240 h. Their sequencing results of 16S rRNA gene amplicons of 66 samples derived from blood, brain, buccal cavity, heart, liver and spleen demonstrated statistically significant time- and organ-dependent changes.

#### 3.2.4. Human Model—Not Only Body Samples

In 2015, Damann et al. [65] analysed bone samples from 12 individuals and three soil samples to assess the effects of decomposition on bacterial communities. They found that partially skeletonized remains maintained a presence of bacteria associated with the human gut, while bacterial composition of dry skeletal remains maintained a community profile similar to soil communities. Differences in microbial profiles among different phases of skeletal decay was greater than the variation within the phases, demonstrating the potential of using bacterial community membership as a temporal benchmark for estimating PMI of skeletonized remains.

Two years later, Adserias-Garriga et al. [66] studied three donated individuals through soil samples around the body taken until advanced decay/dry remains stage. They extracted bacterial DNA, HTS (high-throughput sequencing) techniques were applied and bio-informatic data analysis was performed. At the beginning of the decomposition process the microbiome of the soil was composed of different indigenous soil bacterial communities. As decomposition progresses, Firmicutes community abundance increased in the soil during the bloat stage. They found that the growth curve of Firmicutes from human remains can be used to estimate PMI, in locations and seasons like those of the execution of their experiment.

### 3.3. Human and Animal Models

The only study recovered which considers both human and animal models was conducted by Metcalf et al. [67] in 2015. They made a study using mouse cadavers under controlled laboratory settings and human donors (i.e., people who gave their bodies to science) in outdoor settings. They sampled skin, abdominal cavity and grave soil of five mice for 71 days and skin and grave soil of four human subjects for a maximum of 143 days. Their results show that microbial communities change significantly during decomposition and become more like each other across body sites and grave soils. A Random Forests regression model trained on their microbial data resulted in estimates of the PMI with errors ~2 to 3 days over the first 2 weeks of decomposition.

## 4. Discussion

The main issue in post-mortem microbiology is the dynamic of changes in the composition of the “thanato-microbiome”, conditioned by many endogenous and exogenous factors.

The most accurate estimates of PMI should be provided during the early decay stage of decomposition, in which microbial succession is quite rapid and predictable, but to test this one would need a large amount of data collected from frequent samples taken for a considerable period. It is of utmost importance to understand how to apply this technique wisely, while also considering the best possible way to assist already existing forensic tools, if not to use it in their stead [33].

It is important to consider that it is not always possible to have access to human corpses for research in the post-mortem microbiome field; therefore, most of the studies regarding this branch of science involve animal models (i.e., swine or mice) [34]. Although this may appear as a ‘slowdown’ in the achievement of applicable results, it is in fact an essential step, as only through the study of the microbiome of corpses with known PMI may the construction of more reliable models be allowed. In this setting, the most challenging issue is the interpretation of the results, which calls for the presence of skilled microbiologists and for a great deal of data given that the articles considered demonstrated several differences between animal and human models.

First, mice models can be considered sufficiently standardized since the mice are bred in the laboratory at regulated light, temperature and humidity and fed all in the same way [47]. In order to carry out the experiments, the mice are selected and often chosen to have the same sex, weight and age [51,68], resulting in homogeneous groups, so research designs are more reliable. Furthermore, the time and the cause of death, usually cervical dislocation, in mice models are known with certainty, as animals are sacrificed in the laboratory [51] and thus post-mortem decomposition is also often carried out in a controlled environment. However, in this case, decomposition processes will deviate from the physiological ones that occur in nature in an open environment, where cadaver microbiome is influenced by soil contamination and insects’ colonization. For this reason, some authors have chosen to monitor the progress of the microbiome during the decomposition of corpses buried in the ground in an open space [45,53]. Furthermore, another limitation of the mouse model is that the organism and the composition of its microbiota deviates considerably more from that of humans than other animal models, such as the pig.

Unlike animal models where pre-death conditions are uniform and known, in human models both the conditions of the subject in life and the causes of death can be very different, and this has an impact on the characteristics of the cadaveric microbiome. In fact, it is well known how much the microbiome may differ between individuals depending on various ante-mortem factors i.e., diet, geolocation, lifestyle habits, health status [69,70].

In human models a further consideration concerns the fact that the cause of death and the circumstances in which it occurred also influence the composition of the cadaveric microbiome [62], but in the studies examined in this review this information is often missing and therefore these variables cannot be correlated to the others and to the PMI estimation in the proposed predictive models.

The variation of the microbiome in the various phases of post-mortem phenomena is the crucial element for establishing a reliable predictive model, which should be based on data obtained from conditions that should be as standardized as possible. Therefore, one of the fundamental aspects in determining the PMI using the study of the cadaveric microbiome is represented by the choice of the sampling site and the choice of the best sampling strategy.

Concerning the choice of the sampling site, the studies we considered reflect the trend that research has followed during the last years, that is, sampling both internal and external sites, but each one of them has its own peculiarities, such as being more easily accessible by investigators, a higher reliability degree for certain PMIs and the different ante-mortem microbiome profile depending on the body sites. If we are able to obtain and manage this kind of information, we could have much more data that could help us in assessing PMI more precisely.

In addition to the sampling site and the strategy to be used to carry out the sampling, another critical factor in determining a reliable predictive model is to have a sufficiently large number of data to be able to build an effective, reliable and usable model in the forensic field, where a degree of certainty proof of criminal investigation is required.

Large-scale analysis of microbiome data is necessary to extrapolate information and to create microbiome profiles that could be useful in the forensic context, such as the correlation between changes in microbiome composition and PMI. Machine-learning models generalise data into categories by creating predictions based on input variables [71]. There are several of these, and to assess which one to employ is strictly dependent on the endpoint of the analysis. Most of the articles selected in this review use Random Forest as a machine-learning method, that is, a non-linear model. It consists of a set of decision trees constructed by bootstrapping the dataset and taking model predictions from the trees, which can help reduce generalisation error through multiple subsampling of features.

When considering animal models, after death, the normal intestinal flora present during life is replaced by facultative anaerobic and obligate anaerobic bacteria, as demonstrated by Liu et al. who, by means of a random forest regression model, demonstrated an average error of 20.01 h during 15 days of decomposition [51].

Analysis of different body sites (eyes, ears, nose, mouth, rectum, brain, spleen, liver, heart) revealed that the post-mortem microbiota was associated with reduced taxonomic diversity at external sites. Internal organs were initially sterile, and spleen and liver showed signs of bacterial invasion at 3 days post-mortem, while heart and brain at 10 days post-mortem, Firmicutes and Proteobacteria proved dominant in the composition of the post-mortem microbiota. Furthermore, analysing samples collected at rectum, skin and soil surrounding the corpse, it was found that the more advanced stages of decomposition showed less alpha diversity than the earlier ones [68].

Although in the forensic field the eye has been and is still widely studied, mainly for the estimation of the post-mortem interval [72,73], only one study among those examined in the review included the eye among the sampling sites [57]. This is difficult to explain, as the eye presents itself as an organ that is easily sampled given its location and being relatively anatomically well isolated from the body. The lack of studies in this regard could be motivated by the inherent complexity of this organ, being composed of many different tissues, which would make it difficult to establish an overall microbiome of the eye without analyzing the microbiome of its individual constituents (i.e., iris, cornea, vitreous humor, etc.).

A significant linear relationship between similar microbiome communities and PMI has also been observed. Through Random Forest, Zhang et al. found that the error in predicting PMI from a given microbiome was about 2 days out of 60 days of decomposition, showing less error in soil analysis than skin and rectum [53]. Authors demonstrated that the microbiota of the rectum showed a change in its composition at intervals during 15 days of decomposition, the phylum of Proteobacteria and Firmicutes showed the greatest change during post-mortem and day nine was identified as the turning point [45].

By combining the characterisation of the microbiome of different organs (brain, heart and cecum), significant differences were found between its composition at the time of death and during the following decomposition stages [53]. The best combination was identified in an ANN (artificial neural network) model with the post-mortem caecum microbiome defining an error of 14.5 ± 4.4 h during 15 days of decomposition [53].

A linear regression model relating the relative abundance of oral cavity microbiota and PMI showed that *Gammaproteobacteria* and *Proteus* were best at inferring PMI, especially in the later PMI [44].

Evaluating the oral cavity and rectum showed how during decomposition the bacterial communities of different body sites become more similar to each other. Proteobacteria becomes the predominant phylum in both cavities (oral and rectal), while Firmicutes and Bacteroidetes gradually decrease over time [43]. Considering various organs (intestines, bone marrow, lungs and heart) it was found that organs that are assumed to be sterile during life were colonised by *Clostridium* after death [48]. In a work on soil burial of rats and intestinal sampling, two bacterial taxa were identified: *Enterococcus faecalis* and *Clostridium paraputrificum*, which, investigated for the first time, demonstrated changes in their abundance over time [47].

The changes in the microbial community of the head skin allowed the PMI to be estimated with an error of 3.30 ± 2.52 days over 48 days of decomposition [52].

Considering the literature examined in this review, in animal models variations in Firmicutes and Proteobacteria proved to be useful in estimating PMI, particularly in rectum, eyes, nose, mouth, brain, spleen, liver and heart; in the oral cavity, proteus also proved to be a valid indicator. Moreover, it was noted how the replacement of aerobic bacteria by facultative or obligate anaerobic bacteria often occurs and the microbiome of the soil surrounding the corpse should be analysed, as it may prove more accurate in estimating PMI than the microbiome presents in the organism itself. In addition, several organs that are thought to be sterile in life have shown invasion by *Clostridium* during decomposition.

In addition, in human models different body sites have been studied, in particular, the nasal cavity, the oral cavity, the cecum and its appendix, the sexual organs (uterus and prostate in particular), the skin and the bone tissue.

The analysis of the microbiome of the nasal cavities and the external auditory canal showed that the regression model based on the data set derived from the microbiome of the nose had not yielded significant results, which were instead obtained from the data set consisting of the microbiome of the auditory canal. It was seen that, when considering the microbiome derived from both body sites together, the regression model obtained was more reliable [54].

A change was noted at all body sites from a microbiome consisting mostly of aerobic to anaerobic bacteria [63].

In the oral cavity, Firmicutes and Actinobacteria proved to be predominant in the early post-mortem stages. In the following decomposition stages the predominant families were *Peptostreptococcaceae* and *Bacteroidaceae*, and *Enterococcaceae*. Later, in the bloat stage, *Clostridiales* took advantage of the changes in the environmental conditions and the predominant bacterial families in advanced decomposition stages were *Gammaproteobacteria*, *Pseudomonadaceae*, *Alcaligenaceae* and *Planococcaceae*, which are soil representatives. This precise succession of microbial species in the oral cavity during the stages of decomposition can be used as a predictive model for PMI estimation [55].

In human models, the appendix could be considered an excellent site for intestinal sampling and its microbiota follows a well-defined succession in time during decomposition [62]. The study of the post-mortem changes in the microbial community of the caecum pointed out that bacterial richness increased significantly, while its diversity decreased. The order of *Bacteroidales* decreased significantly while the order of *Clostridiales* increased during decomposition stages [59]. In caecal microbiota, *Bacteroides* and *Lactobacillus* decreased exponentially as PMI increased, while *Bifidobacterium* abundance did not vary significantly and *Bacteroides* and *Lactobacillus* could be used as a quantitative indicator of PMI [58].

Uterus and prostate were found to possess higher alpha diversity than other body sites and demonstrated a significant difference in microbial community composition compared to other non-reproductive organs. The fact that uterus and prostate are relatively more isolated organs than the external environment could justify these findings. Furthermore, uterus and prostate are the last organs to decay during human decomposition, providing some sort of last-resort sampling site in those cases where other organs’ conditions are heavily jeopardised by a long process of decomposition [60]. The human skin microbiome has been found to remain stable during serial sampling over 60 h post-mortem and was like that of living human beings [56].

Deel et al. demonstrated that, in the human bone decomposition process, there is a microbiome core composed mainly of Proteobacteria, which is progressively supplanted by microorganism migration coming from the surrounding environment, i.e., cadaveric skin and soil, resulting in a predictive model for PMI estimation with an accuracy of ±34 days over a time span of 1 to 9 months [61]. Damman et al. had already demonstrated that the partially skeletonised remains retained the presence of gut-associated bacteria after decomposition, while the dry remains possessed a microbial community more similar to that of the surrounding soil [65]. In particular, according to Adserias-Garriga, the soil around the corpse showed, as decomposition progressed, an increase in the abundance of the Firmicutes community during the emphysematous phase, proposing the use of the Firmicutes growth curve as a model to estimate the time since death [66].

Summarizing the results of studies conducted in human models, as regards the sampling site, on several body sites Firmicutes, *Bacteroides* and *Clostridiales* proved to be useful indicators of PMI. The skin microbiome did not prove to be useful in the first few days after death for estimating the post-mortem interval, as it was stable and statistically unchanged from the living microbiome. Analysis of the bone microbiome might be useful in advanced stages of decomposition. Firmicutes should be investigated in the analysis of the microbial community of the soil and oral cavity in the estimation of PMI. The analysis of several body sites at the same time may provide a more accurate and reliable PMI estimation.

Once the usefulness of the various body sampling sites has been understood, depending on the succession of microbial species during decomposition, it is important to evaluate the most correct technique for conducting the sampling and storing the collected biological material. Despite the efforts to use sterile tools for sampling, one of the biggest challenges is obtaining a sample unsullied by other sites’ microbes; therefore, instead of needlessly trying to achieve an uncontaminated sample, it could be more useful to reach an agreement on an acceptable level of sample contamination, which should lead to a basic “white” to be discarded in the interpretation of results. This could be a step in creating a common and standardized methodology for guiding sample collection and analyses [33].

By external body sites we meant those body sites that can be reached and sampled by simple external examination, without the need to dissect, thus, the skin, eyes, and body orifices (oral cavity, nasal cavities, external auditory canals and rectum) may be considered under this category. Otherwise, by internal anatomical sites we meant body sites that require autopsy or biopsies for their sampling, such as thoracic/abdominal organs or bones.

While sampling of external body sites proved to be repeatable over time without compromising or changing the composition of the microbiome given the use of sterile swabs, the major technical problem was encountered when sampling internal organs. In fact, in experiments in which sampling was repeated in the same organ at different time intervals, after the first incision, a mean of contact was created between the sampled organ and adjacent organs or the skin, changing the physiological isolation of the organ from the external environment. This may result in a change from the normal physiological mechanism of tissue decomposition and possible invasion of the organ by microbiota present in adjacent organs, the skin, or the external environment. In order to limit this risk, several authors have proposed procedures to avoid this type of cross contamination. For example, Liu et al. [51] performed the sampling of the cecum of mice under aseptic operative conditions by multiple personnel within a short period of time to avoid the influence of sampling on microbiota variability. Others, such as Heimesaat et al. [46], before mice necropsy disinfected the murine skin/fur with isopropanol solution, in order to significantly reduce surface bacterial loads, thereby minimising the risk of contamination during section. DeBruyn et al., in their human model experiment, made a small incision in the abdomen and used sterile swabs to collect gut material from the caecum. They sealed the incision with tape and re-sampled daily [59]. In the human experiment of Hauther et al. [58] they placed duct tape over the incision in the abdomen and at the next sampling date the original incision was accessed through an incision in the duct tape. Other authors, such as Hyde et al. [63], carried out, in human cadavers, seriate sampling of external sites over time, while sampling internal organs only once at the end of the study time, since accessing the viscera required a destructive procedure of dissection of the abdomen, marking the end of sampling and resulting in a single sampling as opposed to multiple sampling in anatomically external sites. In fact, when a sample is taken from an internal site, the latter is put in communication with the external environment, affecting the evolution of the composition of the microbiota. To partially obviate this problem, a valid option could be to sample two pair organs (e.g., kidneys, ovaries, etc.) at two different times, so that the evolution of the microbiome in the “same” organ can be assessed, with due limitations, at a distance of time, without contaminating it at the time of the first sampling.

Other sampling strategies have been proposed to obtain the best reproducibility of results. For example, Liu et al. [50], instead of sampling the same mouse at different time intervals, divided the total number of mice into the number of samplings they wanted to carry out. In this way, the organs of each animal were sampled only once, without the risk of affecting the succession of changes in the epi-necrotic microorganisms’ community. This model may be considered advantageous for the specificity of the extrapolated results but does not allow the sampling at different post-mortem intervals for the same mouse, following the evolution of the mouse’s microbiome over time. However, this experimental bias could be partially overcome, since in this specific study mice were raised in the same place, reared in a controlled environment and fed in the same way, and this would not be obviously possible in human models, where everyone has different characteristics that are reflected in the composition of the microbiome, even after death.

As far as applicability in the real-world scenario is concerned, we are still a long way from the application of an unambiguous and validated PMI prediction model based on the microbiome. Of the works considered in this review, less than a third have produced a prediction model based on the data they collected. These predictive models are clearly influenced by the small sample size analysed and limited body sites tested. For example, Hu et al. [62] studied the flora of the human appendix for 192 h and found, via a random forest regression model, a mean absolute error of 25.79 ± 0.49 h. Liu et al. [51] studied the murine cecum for 15 day and found, via a random forest regression model, a mean absolute error of 20.01 h. To analyse a longer time span, Deel et al. [61] created a random forest regression model to determine PMI by sampling human bones. Over a study period of 9 months, they predicted PMI with an accuracy of approximately ±34 days. To date, it is difficult to establish which predictive model is the best as there is too much variability between different studies. This variability relates to the study model, the locations sampled and, above all, the time span studied. The choice of biological matrix is also difficult, for the same reasons as above. In order to identify the most accurate predictive model, it is necessary to have several standardized studies investigating several body sites simultaneously in order to compare them with each other, integrating the data, and over ideally the longest possible time span. Given that there is currently no consensus on a single predictive model, the time window in which the microbiome can most help appears to be the late stages of decay, because in the early stages of decay we have several methods, among which the microbiome can act as a support to determinate PMI, while in the long term the classical methods of use are not applicable, so the microbiome could become a benchmark.

Most of the literature studies on PMI estimation using the microbiome use 16S rRNA subunit analysis. New NGS techniques with metagenome sequencing can be applied to reach a deeper level of microbial identification. This, however, requires higher costs, more sophisticated instrumentation and considerable effort in order to have reproducible results. The identification of microbial species, even with more traditional amplicon sequencing, typically requires the comparison of sequence information against a reference genome in a database; however, the discrepancies in the available databases can affect the number and type of microorganisms identified, where unknown microorganisms may not be identified. Single sample studies may be enough to address and identify differences between individuals along with a forensic question, but they usually do not explore more than one piece of metadata variable. Despite that the number of microbiome samples collected are increasing, the great diversity of both methodologies employed for sampling and manners of reporting the data hinder the endeavor of analyzing the information in its wholeness and as a homogeneous entity [74].

In addition to the technical challenges, which need to adapt methods developed and standardized for clinical purposes in the forensic sciences landscape, there are still many uncertainties about the real applicability of these techniques to real casework and in daily forensic practice. Albeit with some limitations that undermine the standardization and dissemination of these techniques, we can hope for the creation of new microbiome databases to be used, first, for research, and subsequently for real applications in the forensic field [75], as for other -omics sciences [76].

These efforts could represent a cornerstone along the road to develop an instrument that gives us the possibility of fast cross checking of microbiomes’ characteristics to facilitate answers, involving PMI assessment, with the degree of certainty and robustness that is required in the investigative and judicial process.

### Study Limitations

A major limitation of this review is the limited number of experimental works dealing with the correlation between PMI and the microbiome, especially considering that the works do not all focus on the human model, but many investigate the animal model. Furthermore, the body sites investigated are diverse, making a uniform interpretation of the data obtained very challenging.

Concerning the 16S sequencing method, the fact that 16S rRNA genes are housekeeping ones carries some inherent limitations, such as the impossibility to assess the characteristics of these bacteria (e.g., metabolic potential, antibiotic resistance, virulence, etc.) or the total microbial load (which would require quantitative sequencing methods) [77]. The number of OTUs generated for a sample is also significantly affected by which variable region is selected for targeted sequencing, thus, as a new standard, full 16S rRNA gene sequencing was proposed [78]; still, despite that full sequencing is becoming more and more popular, the targeted sequencing of 16S rRNA variable region remains the more widespread method applied [40].

## 5. Conclusions

In this review, the most frequently tested sites for microbiome analysis by means of 16S rRNA sequencing were evaluated; these studies, conducted on both animal and human models, all yielded results that were promising in their own way, but were not yet sufficient to routinely include microbiome analysis within validated protocols for PMI estimation in real casework and criminal investigations. For this reason, more efforts should be put in place on human models, taking into account all influencing factors in microbiome changes after death, namely environmental and individual variables, including those not considered in the works reviewed such as the cause of death and living health conditions. It would also be desirable to focus on a smaller number of body sites, validated as more reliable, in order to obtain a large amount of data per single body district and thus make it possible to create more specific reference datasets.

## Figures and Tables

**Table 1:** Summary of the main characteristics of the studies included in the review. M: male/s, F: female/s, N.A. not available.

Reference	Model	Body Site	Numerosity (Number of Subjects Enrolled in the Study)	Temperature	Time Interval
Liu R. et al., 2021	Animal (murine)	Cecum	24	25 ± 1.5 °C	15 days
Hu L. et al., 2021	Human	Vermiform appendix, transverse colon	63 (45 M + 18 F)	1 °C	8 days
Deel H. et al., 2021	Human	Bones	6	0 °C	9 months
Zhang J. et al., 2021	Animal (murine)	Rectum, skin, grave soil	50 M	N.A.	60 days
Li H. et al., 2021	Animal (murine)	Rectal	8 M	21.63 ± 0.93 °C	15 days
Lutz H. et al., 2020	Human	Brain, heart, liver, spleen, prostate, uterus	40 (26 M + 14 F)	1 °C	24–432 h
Pittner S. et al., 2020	Human	Eyes, ears, mouth, nose, rectum, skin	2 M	From 1.4 to 34.6 °C	16–122 days
Liu R. et al., 2020	Animal (murine)	Brain, heart, cecum	80	25 ± 1.5 °C	15 days
Dong K. et al., 2019	Animal (murine)	Oral cavity	24	22.4 °C	240 h
Burcham Z.M. et al., 2019	Animal (murine)	Heart, stomach, intestines, bone marrow	45	N.A.	170 h
Burcham Z.M. et al., 2019	Animal (murine)	Heart, intestines, bone marrow, lungs	90	N.A.	30 days
Kodama W.A. et al., 2019	Human	Skin	16 (11 M + 5 F)	6 °C	60 h
Iancu L. et al., 2018	Animal (murine)	Intestinal	60	From 7.28 to 25.37 °C	30 days
Adserias-Garriga J. et al., 2017	Human	Soil sample around the body	3 (1 M + 2 F)	From 21 to 27 °C	12 days
Adserias-Garriga J. et al., 2017	Human	Oral cavity	3 (1 M + 2 F)	From 21 to 27 °C	12 days
DeBruyn J.M.et al., 2017	Human	Cecum	4	N.A.	7 days
Johnson H.R. et al., 2016	Human	Ear, nose	21	3 °C	24 h
Javan G.T. et al., 2016	Human	Blood, brain, buccal cavity, heart, liver, spleen	27 (15 M + 12 F)	1 °C	3.5–240 h
Guo J. et al., 2016	Animal (murine)	Buccal cavity, rectum	18 F	From 22.71 to 27.67 °C	8 days
Metcalf J.L. et al., 2016	Animal (murine) and human	Skin, abdominal cavity, grave soil	5 rats—4 humans	N.A.	71–143 days
Hauther K.A. et al., 2015	Human	Cecum	12	0 °C	20 days
Damann F.E. et al., 2015	Human	Bones, soil	12	N.A.	24–1692 days
Hyde E.R. et al., 2013	Human	Mouth, rectal, intestine, stomach	2	From 17 to 3.33 °C	>102 days
Metcalf J.L. et al., 2013	Animal (murine)	Abdominal cavity, skin, grave soil	40	N.A.	48 days
Heimesaat M.M. et al., 2012	Animal (murine)	Intestine	N.A.	21 °C	72 h

## Data Availability

No publicly archived datasets were created. Data are available upon request to the corresponding Authors.
